# Viral infection of an estuarine *Synechococcus* influences its co-occurring heterotrophic bacterial community in the culture

**DOI:** 10.3389/fmicb.2024.1345952

**Published:** 2024-01-25

**Authors:** Hongcong Man, Binbin Liu, Hongrui Zheng, Jihua Liu, Yongle Xu, Feng Chen

**Affiliations:** ^1^Institute of Marine Science and Technology, Shandong University, Qingdao, China; ^2^Institute of Marine and Environmental Technology, University of Maryland Center for Environmental Science, Baltimore, MD, United States

**Keywords:** *Synechococcus*-heterotroph coculture, cyanophage infection, microbial dynamics, bacterial community transition, bacterial interaction, nutrient cycling

## Abstract

Viruses are infectious and abundant in the marine environment. Viral lysis of host cells releases organic matter and nutrients that affect the surrounding microbial community. *Synechococcus* are important primary producers in the ocean and they are subject to frequent viral infection. In the laboratory, *Synechococcus* cultures are often associated with bacteria and such a co-existence relationship appears to be important to the growth and stability of *Synechococcus*. However, we know little about how viral lysis of *Synechococcus* affects the co-existing bacteria in the culture. This study investigated the influence of viral infection of *Synechococcus* on co-occurring bacterial community in the culture. We analyzed the community composition, diversity, predicted functions of the bacterial community, and its correlations with fluorescent dissolved organic matter (FDOM) components and nutrients after introducing a cyanophage to the *Synechococcus* culture. Cyanophage infection altered the bacterial community structure and increased the bacterial diversity and richness. Increased bacterial groups such as *Bacteroidetes* and *Alphaproteobacteria* and decreased bacterial groups such as *Gammaproteobacteria* were observed. Moreover, cyanophage infection reduced bacterial interactions but enhanced correlations between the dominant bacterial taxa and nutrients. Unique FDOM components were observed in the cyanophage-added culture. Fluorescence intensities of FDOM components varied across the cyanophage-infection process. Decreased nitrate and increased ammonium and phosphate in the cyanophage-added culture coupled with the viral progeny production and increased substance transport and metabolism potentials of the bacterial community. Furthermore, increased potentials in methane metabolism and aromatic compound degradation of the bacterial community were observed in the cyanophage-added culture, suggesting that cyanophage infections contribute to the production of methane-related compounds and refractory organic matter in a microcosm like environment. This study has the potential to deepen our understanding of the impact of viral lysis of cyanobacteria on microbial community in the surrounding water.

## Introduction

Microorganisms play a crucial role in driving biogeochemical processes in the ocean (Singh et al., 2010; Liu et al., 2019). The phytoplankton are the basis of the marine food web (Azam et al., 1983; Buchan et al., 2014; Liu et al., 2019). Phytoplankton release dissolved organic matter (DOM) as a by-product of photosynthetic carbon fixation, contributing significantly to the bioavailable organic carbon and nitrogen in the ocean (Nagata and Kirchman, 1992; Kaiser and Benner, 2008; Fiore et al., 2015). The labile DOM is subsequently assimilated by heterotrophic bacteria and mineralized into inorganic nutrients, which could be further absorbed by primary producers. The transition of the phytoplankton community usually leads to the metabolite change in the environment and thus affects the community of surrounding heterotrophic bacteria (Ferrer-González et al., 2023). Additionally, viruses are important members of the microbial entity and are also deemed significant contributors to global biogeochemical cycles. It is estimated that 10^28^ viral infections occur in the ocean each day (Suttle, 2007). Viral lysis of microorganisms rapidly releases intracellular substances, cell debris, and progeny virus particles into the environment, which contributes to the DOM pool in the ocean (Suttle, 2007) and directly or indirectly influences the microbial population dynamics and community structure (Proctor and Fuhrman, 1990).

Marine picocyanobacteria, mainly composed of *Prochlorococcus* and *Synechococcus*, are the most abundant and widely distributed phytoplankton in the ocean (Scanlan, 2012), contributing up to 50% of the primary production in the ocean (Goericke and Welschmeyer, 1993). Approximately 15% of cyanobacteria in the ocean are infected by viruses (Proctor and Fuhrman, 1990). It is well known that cyanophages usually encode various auxiliary metabolism genes (AMGs), the expression of which would reprogram the host’s central metabolism during infection (Sullivan et al., 2010; Puxty et al., 2015). Compared to normal cells, infected cells contain more sugars, amino acids, lipids, and nucleotides (Jacobson et al., 2021). It is reported that the DOM composition of marine *Synechococcus* viral lysates differs from that of normal cell exudates and mechanical lysates (Jover et al., 2014; Xiao et al., 2021). Viral infection results in an increase in the nitrogen content of the *Synechococcus* viral lysates (Zhao et al., 2019; Zheng et al., 2021a). Virus-mediated picocyanobacterial lysates constitute a significant portion of the rapidly cycled carbon in the surrounding environment and can be utilized by heterotrophic bacteria (Bratbak et al., 1992; Gobler et al., 1997; Middelboe et al., 2003).

Recently, the influences of picocyanobacteria-derived DOM or viral lysates on marine bacterial communities have been frequently studied. A study investigating the response of bacterial community in a coastal seawater microcosm to the *Synechococcus*-derived DOM demonstrates an immediate rise of *Alphaproteobacteria* and *Flavobacteria* (Xie et al., 2020; Wang et al., 2022). Additionally, increased relative abundances of *Gammaproteobacteria*, *Betaproteobacteria*, and *Flavobacteria* are induced in another coastal bacterial community by the addition of *Synechococcus* viral lysates to the microcosmic sea water (Zhao et al., 2019). Furthermore, the growth of *Alteromonadales* in an oligotrophic bacterial community is stimulated by the introduction of *Prochlorococcus* viral lysates and becomes the dominant taxa within 1 day (Xiao et al., 2021). However, the impact of *in situ* viral infection of picocyanobacteria on the transition of the surrounding bacterial community in marine environments remains poorly understood.

Marine picocyanobacterial isolate cultures (including *Synechococcus* and *Prochlorococcus*) usually contain heterotrophic bacteria (Moore et al., 2007; Cole et al., 2014; Zheng et al., 2018, 2021b; Cruz and Neuer, 2019), which may be grabbed from the natural habitat during the picocyanobacterial isolation (Zheng et al., 2021a). Many picocyanobacteria grow more efficiently when cocultured with heterotrophic bacteria instead of being in axenic cultures (Hayashi et al., 2011; Shen et al., 2011; Cole et al., 2014). The co-occurring heterotrophic bacteria are thought to reduce the surrounding oxidative stress, provide vitamins, and facilitate the recycling of inorganic nutrients required for picocyanobacterial growth (Morris et al., 2008, 2011). In laboratory picocyanobacterial cultures, the dominant populations of the co-occurring bacterial community are composed of *Alphaproteobacteria*, *Gammaproteobacteria*, and *Bacteroidetes*, which are congruent with those found in the world’s oceans (Kirchman, 2002; Zheng et al., 2018). It is reported that, among the co-occurring heterotrophic bacteria in a *Synechococcus* culture, specific species of *Alphaproteobacteria* and *Flavobacteria* are responsible for the organic compound transformation from high molecular weight (HMW) to low molecular weight (LMW) (Zheng et al., 2020), which is also congruent with the rapid rise of *Alphaproteobacteria* and *Flavobacteria* in a coastal bacterial community in response to the introduction of *Synechococcus*-derived DOM which is rich in HMW molecules (Xie et al., 2020; Wang et al., 2022). Therefore, picocyanobacteria–heterotroph cocultures can be used as model microcosms to simulate and investigate microbial interactions in the natural marine environment (Zheng et al., 2021b).

It is thought that the biochemical impact of virus invasion on the host is not limited to lysing the cell, but begins from the moment the virus genetic material enters the cell (Zimmerman et al., 2020). We hypothesize that viral infection affects the surrounding microbial community and element cycling in the marine environment once upon the viral entry into the host cell, rather than until the virus lyses the host cell. To test this hypothesis, in this study, we investigated the co-occurring heterotrophic bacterial community transition in an estuarine *Synechococcus* culture in response to the introduction of a cyanophage that infects the *Synechococcus* strain and analyzed the potential microbial metabolic changes induced by bacterial community transition to illustrate the impact of *in situ* viral infection of picocyanobacteria on the surrounding bacterial community in the marine environment.

## Materials and methods

### Experimental setup and sample collection

Estuarine *Synechococcus* strain CBW1101 was cultured in SN medium (Waterbury et al., 1986) with a salinity of 15 ‰ at 22°C under a constant light intensity of 20 μmol photon·m^−2^·s^−1^. To investigate the response of the co-occurring heterotrophic bacterial community to the introduction of a cyanophage in the *Synechococcus* culture, a total of 3 L of exponentially growing culture of CBW1101 (OD_750_ = 0.4) was split equally into six flasks, three of which were inoculated with a cyanophage, S-CBWM1, at a multiplicity of infection (MOI) of 3, and the other three were used as controls. Both cyanophage-inoculated and control flasks were incubated at 22°C under a constant light intensity of 20 μmol photon·m^−2^·s^−1^ in a shaker at a speed of 120 rpm. Subsamples for abundance determination of *Synechococcus* cells, heterotrophic bacterial cells and cyanophage particles were collected on days 0, 1, 2, 3, 4, 5, 6, and 7. Subsamples for heterotrophic bacterial community structure, fluorescent DOM (FDOM) composition, and nutrients determination were collected on days 0, 2, 4, and 6. For microbial abundance counting, 2 mL of each culture was taken and fixed with glutaraldehyde at a final concentration of 1% v/v for 15 min in the dark. After snap freezing in liquid nitrogen, the abundance samples were kept at −80°C until flow cytometry analyses. To collect heterotrophic bacterial community samples, 10 mL of liquid culture was filtered through 0.22-μm-pore-size polycarbonate filters (Millipore) at a pressure of <0.03 MPa. The filters were flash-frozen in liquid nitrogen and stored at −80°C until DNA extraction. Fifteen milliliters of each culture were filtered through a 0.22-μm-pore-size filter (Millipore), and the filtrates were collected and stored at −20°C for nutrient analysis.

### Abundance determination of *Synechococcus*, heterotrophic bacteria, and cyanophage

The abundances of *Synechococcus*, heterotrophic bacteria, and cyanophage were enumerated using a flow cytometer (Epics Altra II, Beckman Coulter, USA) according to the method described as previously (Liang et al., 2014; Cai et al., 2015; Zheng et al., 2018). Briefly, the frozen samples were thawed at 37°C and diluted with Tris-EDTA buffer (pH = 8, Sigma-Aldrich, Darmstadt, Germany) into an appropriate concentration that is easy for flow cytometry analyses. The prokaryote samples were stained with SYBR Green I (Invitrogen) at a final concentration of 0.01% v/v for 15 min in the dark at room temperature. The cyanophage samples were stained with SYBR Green I (Invitrogen) at a final concentration of 0.005% v/v for 10 min at 80°C in the dark and cooled at room temperature for 5 min. The microbial abundance data was acquired and analyzed by the EXPOTM 32 MultiCOMP flow cytometry analysis software and FCM Express software (Cai et al., 2015; Zheng et al., 2021b). The abundance of *Synechococcus* was calculated according to the method described by Jiao et al. (2002), and cells were detected from plots of side scatter versus red fluorescence signals and orange fluorescence versus red fluorescence signals (Jiao et al., 2005; Zheng et al., 2018, 2021b; Wang et al., 2019). Prokaryote cells counting was performed according to the method described by Marie et al. (1999) (Liang et al., 2014), and cells were enumerated in plots of side scatter versus green fluorescence signals and red fluorescence versus green fluorescence signals (Zheng et al., 2018, 2021b; Wang et al., 2019). The abundance of heterotrophic bacteria is calculated by subtracting the abundance of *Synechococcus* from the abundance of prokaryote cells mentioned above. The cyanophage abundance was determined according to the method of Brussaard (2004) (Liang et al., 2014) and discriminated on the basis of green fluorescence and side scatter signals (Marie et al., 1999; Jiao et al., 2005; Wang et al., 2019; Zheng et al., 2021b).

### DNA extraction

The microbial DNA was extracted using the phenol-chloroform-isoamyl alcohol method referring to the method on^1^, and the method as described previously (Wang et al., 2017). Briefly, filters were first fragmented and placed into 2 mL sterile tubes along with sucrose lysis solution. After a freeze-thawing process repeated three times, these tubes were then incubated at 37°C for 1 h. Afterward, 5 μL of lysozyme (with a final concentration of 150 μg mL^−1^) was added and incubated at 37°C for 1 h. The supernatant was collected and then treated with Protease K (final concentration of 100 ug mL^−1^) and 10% SDS (final concentration of 1% w/v). Subsequently, 0.05 volumes of 5 M NaCl were added to the supernatant, and the mixture was subjected to a water bath for 2 h at 55°C. Then, an aqueous phenol extraction and an extraction with phenol/chloroform were performed. Following these extractions, the supernatant was precipitated with 0.8 volumes of isopropanol for 24 h. The resulting DNA pellet was subsequently washed with precooled 70% ethanol. Finally, the extracted DNA was re-suspended in 50–100 μL of sterilized water and stored at −80°C until further use.

### Sequence generation and processing

The 16S rDNA V3-V4 hypervariable region was used to investigate the microbial community structure of each DNA sample. The V3–V4 rRNA target region was amplified using the forward primer 338F (5’-ACTCCTACGGGAGGCAGCA-3′) and the reverse primer 806R (5’-GGACTACHVGGGTWTCTAAT-3′) (Wear et al., 2018). Subsequently, the amplicons were sequenced with an Illumina Hiseq2500 platform. Demultiplexing and quality filtering of raw sequences were performed using QIIME2 (Bolyen et al., 2019). VSEARCH 2.7.1 was used to remove chimeras and cluster high-quality reads to generate operational taxonomic units (OTUs) with a 0.97 sequence identity threshold. Additionally, sequences assigned to archaea and cyanobacteria were removed to refine the dataset. To avoid potential bias caused by varying sequencing depth, sequencing data for each sample were normalized by the smallest sample size. Rarefaction curves (Supplementary Figure S1) were generated based on the OTU richness of each sample. A variety of alpha diversity indexes, such as Shannon, Simpson, and Chao1 index, were computed using the vegan package in R version 4.2.1. The *t*-test was performed to compare the difference in alpha diversity indexes and bacterial taxa between the cyanophage-added and the control groups, was performed on the Tutools platform^2^. Phylogenetic Investigation of Communities by Reconstruction of Unobserved States (PICRUSt) was employed to predict the functional traits of bacterial communities. Furthermore, the relative abundances of KEGG pathways were compared between the control and cyanophage-added groups using the OmicStudio tools^3^. Clustering Spearman correlation heatmaps were generated using the OmicStudio tools (see text footnote 3).

### DOM fluorescence measurements and parallel factor modeling analysis

Excitation emission matrix (EEM) spectra of DOM samples were obtained using a Cary Eclipse (Agilent, USA) fluorimeter, with excitation ranging from 250 to 600 nm at 1-nm intervals and emission ranging from 250 to 800 nm at 2-nm intervals. Subsequently, the ultrapure Milli-Q water EEM spectra were used to correct the blank and Raman-normalize the EEMs of the samples. The EEM spectra were then modeled using parallel factor (PARAFAC) analysis with MATLAB 2020a and the DOMFluor toolbox (Stedmon and Bro, 2008). The fluorescence intensity of each component was assessed using the maximum fluorescence. A t-test was conducted on the Tutools platform (see text footnote 2) to compare the difference of components between the cyanophage-added and the control groups. Furthermore, redundancy analysis (RDA) was performed using the OmicStudio tools (see text footnote 3) to examine the influence of FDOM components on bacterial communities.

### Nutrient determination and analysis

Nutrient concentrations, including NO_3_^−^, NH_4_^+^, and PO_4_^3−^, were measured using a segmented flow analyzer (SEAL Analytical Ltd., AA3 HR Autoanalyzer) as described previously (Ren et al., 2022). The *t*-test was performed to compare the difference of nutrients between the cyanophage-added and the control groups on Tutools platform (see text footnote 2). RDAanalysis was conducted using the OmicStudio tools (see text footnote 3) to investigate the influence of nutrients on bacterial communities. Clustering heatmaps based on the Spearman correlation analysis between the top six genera and the nutrients were generated using the OmicStudio tools (see text footnote 3).

### Microbial network analysis

All possible Spearman’s rank correlations were calculated between the relative abundance of OTUs with R (version 4.2.1). The correlation with a statistically significant *p* value of <0.05 was selected and presented. The networks of the cyanophage-added and the control groups were displayed separately with Cytoscape 3.9.1 (Zhao et al., 2019). Basic indexes (average degree, edge, node, graph density, and others) were calculated with the network analyzer in Cytoscape 3.9.1.

## Results and discussion

### Cyanophage infection dynamics and its impact on the heterotrophic bacterial dynamics in the culture

**Figure 1:**
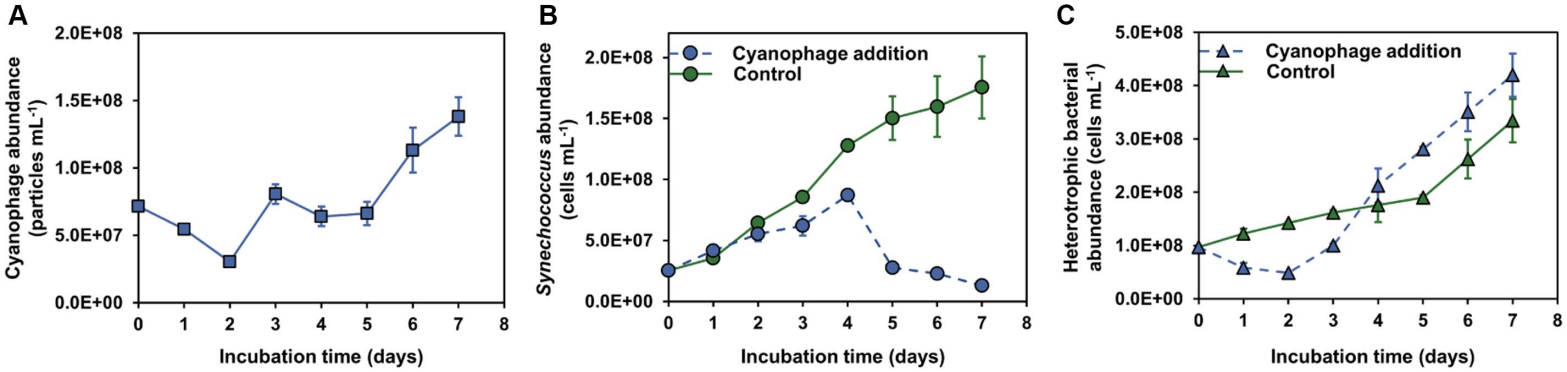
Microbial dynamics over the incubations. **(A)** Cyanophage abundance; **(B)** Synechococcus abundance; **(C)** Heterotrophic bacterial abundance.

To investigate the impact of *in situ* cyanophage infection on the heterotrophic bacterial community co-occurring in the *Synechococcus* culture, *Synechococcus* strain CBW1101 and cyanophage S-CBWM1, which were isolated from the same source of Chesapeake Bay (Xu et al., 2015, 2018), were employed in this study. After addition to the CBW1101 culture, the abundance of S-CBWM1 decreased gradually in the first 2 days, indicating the phage adsorption and entry into the host cells (Figure 1A). Then, the S-CBWM1 abundance began to increase and reached the first peak on day 3 and the second peak on day 7, which corresponded to the growth inhibition beginning on day 2 and the subsequent significant decrease of the CBW1101 abundance beginning on day 4 (Figures 1A,B). Accordingly, S-CBWM1 infection was in the latent period during the initial 2 days and began to lyse the host cells around day 2. Subsequently, the released progeny cyanophages re-adsorbed to the host cells and initiated a second round of lifecycle. It is reported that in laboratory *Synechococcus* cultures, cyanophage addition results in infections of no more than 50% of the *Synechococcus* cells, even when viral abundances exceed those of their hosts (Deng et al., 2012). Therefore, it is reasonable to see that even though S-CBWM1 was added at a MOI of 3, not all CBW1101 cells were infected and lysed during the initial round of infection, which allowed the occurrence of the second round of infection.

The dynamics of *Synechococcus* and cyanophage showed three distinct phases of the infection (Supplementary Figure S2). The first phase (0–2 d) represented the initial round of infection. The second phase (2–4 d) indicated the occurrence of the first round of cell lysis followed by the second round of infection. Compared to the control group, the 4.1 × 10^7^ cells mL^−1^ lower CBW1101 abundance of the cyanophage-added group on day 4 could be attributed to the first round of cell lysis. The third phase (4–7 d) represented the second round of cell lysis. Notably, the fourth day marked the time point with the highest number of infected cells. Additionally, the *Synechococcus* abundance on day 6 had declined to 26% of the level on day 4 due to the viral lysis.

**Figure 2:**
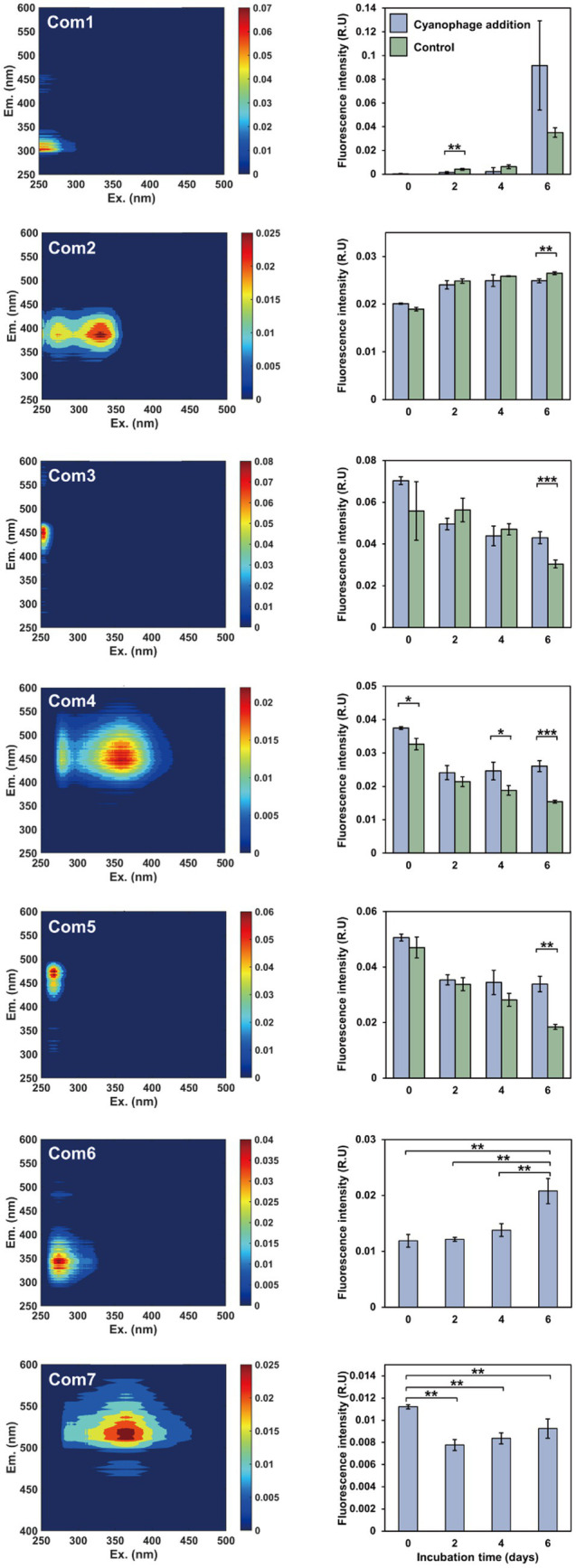
The FDOM components and fluorescent intensity variations during the incubations. Left panels, excitation-emission matrix contours of seven FDOM components; right panels, the fluorescent intensity variations of the FDOM components over the incubation.

**Figure 3:**
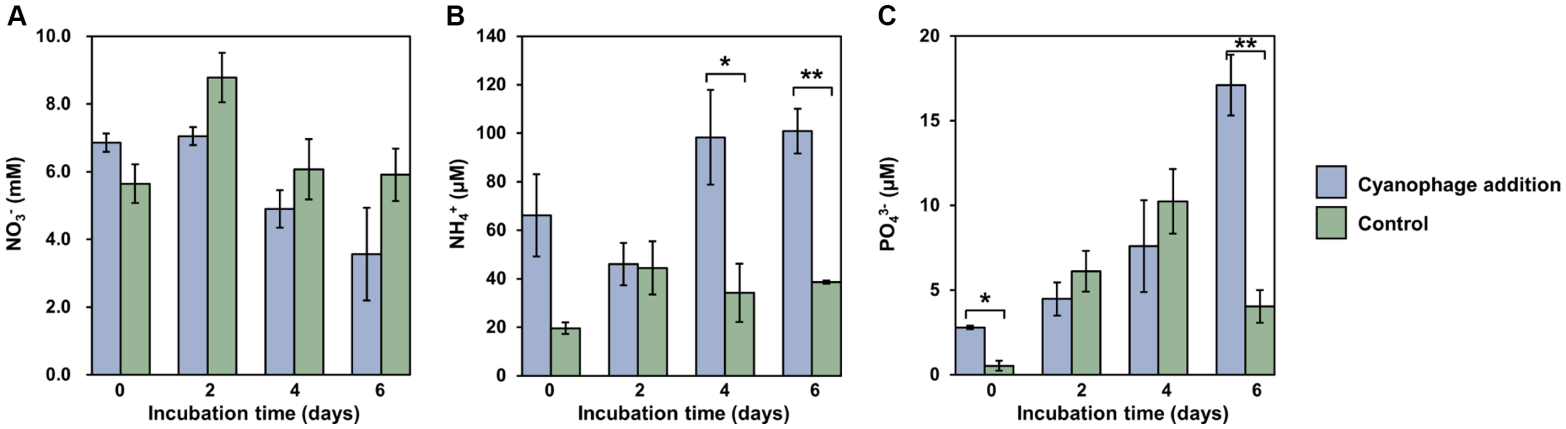
Nutrient variations over the incubation. Level of significance: *p < 0.05; **p < 0.01. **(A)** NO_3_^-^; **(B)** NH_4_^+^; **(C)** PO_4_^3-^.

After cyanophage addition, the growth of heterotrophic bacteria was inhibited in the first phase of infection and then stimulated at a greater rate than that in the control, with the bacterial abundance exceeding that of the control on day 4 (Figure 1C). In the first phase, cyanophage infections of *Synechococcus* were in the latent period, suggesting that viruses can take over the control of host activity, a scenario similar to what has been reported in other studies (Waldbauer et al., 2019; Warwick-Dugdale et al., 2019). The growth inhibition of heterotrophic bacteria in the first phase can be attributed to the disparity in exudates released by the infected and normal cells and nutrient level variations (Figures 2, 3). The increased growth rate of heterotrophic bacteria after day 2 may be stimulated by the viral lysis of *Synechococcus* cells. The impact of cyanophage infection on the bacterial dynamics revealed that viral influence on both the host and the surrounding ecosystem is not solely at the time of host lysis, but rather occurs upon phage entry into the host (Rosenwasser et al., 2016; Zimmerman et al., 2020).

### Impact of cyanophage infection on the FDOM production and nutrient cycling

The DOM optical property analysis was used to characterize the DOM compositions in the cyanophage-added and control cultures. Through PARAFAC analysis, a total of seven fluorescent components were identified, comprising two protein-like components and five humic-like components (Figure 2, Supplementary Figure S3). The first five components were shared between the cyanophage-added and control cultures, whereas the remaining two components were exclusive to the cyanophage-added culture. Components 1 and 6 (Com1 and Com6) were identified as amino acid-like DOM, with Com1 similar to a tyrosine-like peak B and Com6 similar to a tryptophan-like peak T (Stedmon and Bro, 2008). Components 2–5, and 7 (Com2–5, and Com7) belonged to humic-like FDOM. Com2 was similar to the marine humic-like peak M (Kothawala et al., 2012). Com3 can be categorized as a terrestrial humic-like peak A (Stedmon and Bro, 2008). Com4 was similar to a combination of terrigenous humic-like peaks A and C, while Com5 resembled the humic-like peak A (Stedmon and Bro, 2008).

Significant differences in FDOM components were observed between the cyanophage-added and the control cultures in different phases (Figure 2, right panel). On day 2, the fluorescence intensity of Com1 in the cyanophage-added culture was significantly lower than that in the control, which indicated that cyanophage-hijacked *Synechococcus* cells reduced the secretion of Com1 or the changed bacterial community increased the utilization of Com1 in phase I. On day 4, Com4 showed significantly higher fluorescence intensity in the cyanophage-added culture than in the control. On day 6, Com2–5 exhibited significant differences between the cyanophage-added and the control cultures. The fluorescence intensity of Com2 in the cyanophage-added culture was significantly lower than that in the control. Conversely, the fluorescence intensities of Com3–5 in the cyanophage-added culture were significantly higher than those in the control. The accumulation of Com2 in the control culture suggested its production by *Synechococcus* and its resistance to bacterial utilization. The consistent levels of Com2 in the cyanophage-added culture from day 4 to 6 can be attributed to the substantial reduction of *Synechococcus* cells and the cessation of Com2 production in phase III. The fluorescence intensity of Com3–5 in the control culture gradually decreased over time, implying the bioavailability and consumption of these components by the bacterial community. Whereas in the cyanophage-added culture, the fluorescence intensity of Com3–5 remained relatively stable from day 2, indicating an increase in production of these components by cyanophage-infected *Synechococcus* cells or a decrease in consumption of these components due to releases of other more labile DOM during cyanophage infection and transition in the bacterial community. Although the fluorescence intensity of Com2 on day 6 did not show a significant difference between the cyanophage-added and control cultures, they are notably higher than those observed in phase I and II. The substantial increase of Com2 in the cyanophage-added culture on day 6 may be attributed to the viral lysis of *Synechococcus* cells. The initial presence of Com6 and Com7 in the cyanophage-added culture at the onset of the experiment may be attributed to the introduction of cyanophage suspensions. The subsequent gradual accumulation and the notable increase on day 6 indicated that Com6 primarily originated from the viral lysis of *Synechococcus* cells. Despite the decline in fluorescence intensity of Com7 during phase I, likely due to bacterial consumption, the gradually increased fluorescence intensity of Com7 from day 2 indicated its production through viral infection of *Synechococcus* cells. Although FDOM only represents a small fraction of DOM, the various components and their fluorescence intensity changes suggested that viral infection of *Synechococcus* altered the DOM composition in the culture. The FDOM components in the cyanophage-added group exhibited significant differences from those of the control in different phases. Furthermore, different FDOM compositions in three phases also indicated the disparity between the virocell exudates and viral lysates.

Viral infection significantly affected the nutrient dynamics within the *Synechococcus*–heterotrophic bacteria coculture system (Figure 3). The introduction of cyanophages that were suspended in viral lysates caused relatively higher levels of nutrients at the onset of the experiment (Figure 3). Different from the generally stable levels of nitrate in the control, the nitrate concentration in the cyanophage-added cultures decreased gradually from day 2 and was significantly lower than those in the control (Figure 3A), which can be attributed to the increased nitrogen demand of infected *Synechococcus* cells for the synthesis of phage progeny. As a result, inorganic nitrogen assimilated during infection may be converted into organic nitrogen in the form of phage particles (Waldbauer et al., 2019). Moreover, the cyanophage-added cultures consistently exhibited a higher concentration of ammonium than the control throughout the incubation. Especially on day 4 and 6, the cyanophage-added group demonstrated a significant increase in the ammonium concentration, reaching approximately three times of those in the control (Figure 3B). Furthermore, there was a significant accumulation of phosphate in the cyanophage-added culture, particularly on day 6. Following extensive cell lysis, the phosphate concentration in the cyanophage-added culture was approximately four times higher than that in the control (Figure 3C). The elevated concentrations of ammonium and phosphate during the viral infection may be the results of the bacterial mineralization of the *Synechococcus* viral lysates (Zhao et al., 2019; Zheng et al., 2020, 2021b), which corresponds to significantly higher bacterial abundances in the cyanophage-added group than those in the control from the fourth day (Figure 1C). During phase III, viral infection led to the lysis of a large number of *Synechococcus* cells, which may release various organic compounds such as carbohydrates, cell wall glycans, lipids, phycobilisomes, and proteins, etc. (Zhao et al., 2019). These organic compounds were ultimately converted into the inorganic form of ammonium and phosphates through bacterial mineralization. Consequently, viral infection not only modified the composition and diversity of DOM, but also influenced the conversion of nutrients between organic and inorganic forms. Specifically, the viral infection of *Synechococcus* led to the conversion of inorganic nitrogen of nitrate into organic nitrogen mainly composed of cyanophage particles. Conversely, the considerable quantity of organic matter released through viral lysis could be transformed into inorganic ammonium and phosphate by heterotrophic bacteria, promoting the growth of the surrounding phytoplankton and re-entering the microbial loop in the natural environment.

### Cyanophage infection increased the diversity and richness of the heterotrophic bacterial community

The transition of the heterotrophic bacterial community after the cyanophage addition was monitored to investigate the influence of cyanophage infection on the surrounding heterotrophic bacteria. A total of 514,434 16S rRNA gene sequences were obtained from eight heterotrophic bacterial communities on days 0, 2, 4, and 6, ranging from 17,976 to 27,923 per sample in the cyanophage-added group and 19,176 to 28,921 in the control. These sequences were grouped into 67 OTUs at 97% identity, with 16 to 19 OTUs identified in samples of the cyanophage-added group and 14 to 21 OTUs identified in samples of the control.

**Figure 4:**
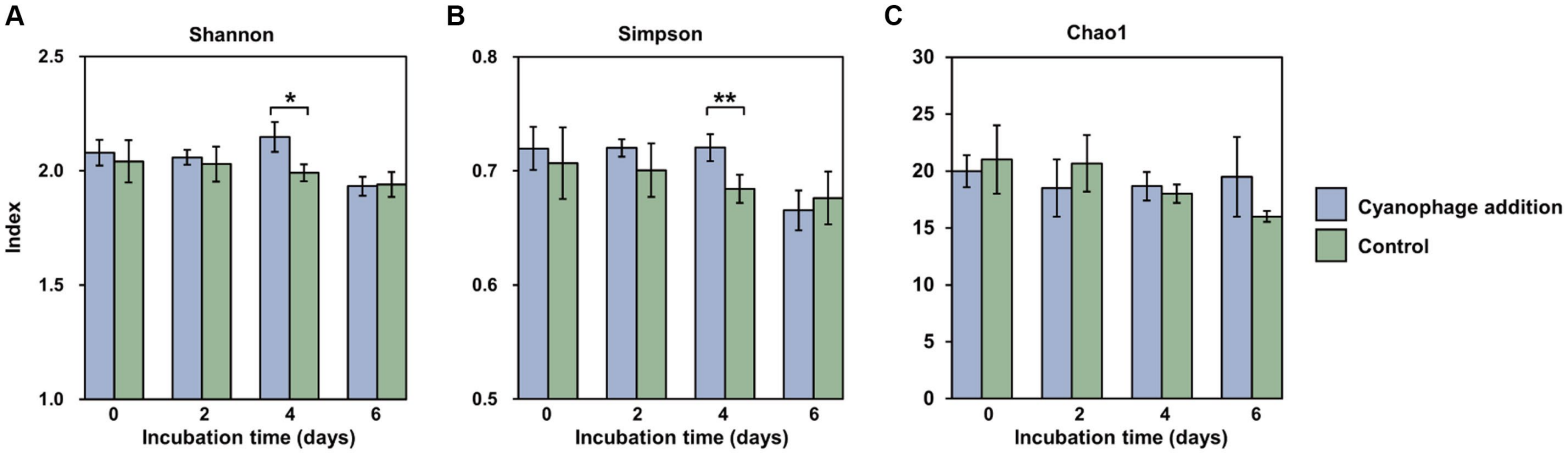
Shannon **(A)**, Simpson **(B)** and Chao1 **(C)** indexes at the OTU level in the cyanophage-added (blue) and the control (green) groups over the incubations. Level of significance: *p < 0.1; **p < 0.05.

As the “kill the winner” agent, virus-induced mortality can be considered a potential mechanism for augmenting the evenness of the bacterial community and ultimately enhancing bacterial diversity (Thingstad and Lignell, 1997; Weinbauer et al., 2007). The *α*-diversity of the bacterial community at the OTU level in this study further supports this opinion. The Shannon (*t*-test, *p* = 0.058) and Simpson (*t*-test, *p* = 0.041) indexes in the cyanophage-added group demonstrated a statistically significant increase compared to those in the control on day 4 (Figures 4A,B), when the *Synechococcus* cell lysis induced by the first round of infection had been started for 2 days and the second round of infection had also been initiated. In addition, different from the gradually decreased Chao 1 index observed in the control (Figure 4C), the Chao 1 index in the cyanophage-added group remained consistent during the whole incubation time, suggesting that the viral infection of *Synechococcus* maintained the richness level of the bacterial community.

It is noteworthy that although the maximum *Synechococcus* cell lysis occurred on day 5 and 6, the most significant influence of viral infection on the diversity of heterotrophic bacterial community was observed on day 4 rather than on day 6. Since the DOM is thought to be the primary determinant of bacterial diversity, it can be inferred that viral lysates from the first round of infection together with exudates released by infected *Synechococcus* cells of the second round of infection on day 4 sustained a more diverse bacterial community than viral lysates did, which were the main DOM source on day 6. Moreover, exudates of infected cells facilitated the growth of different bacterial taxa from those supported by viral lysates, which further emphasizes the importance of infected cells, which are also referred to as virocells, in marine ecosystems (Rosenwasser et al., 2016; Zimmerman et al., 2020).

### Influence of the viral infection of *Synechococcus* on the heterotrophic bacterial community composition

**Figure 5:**
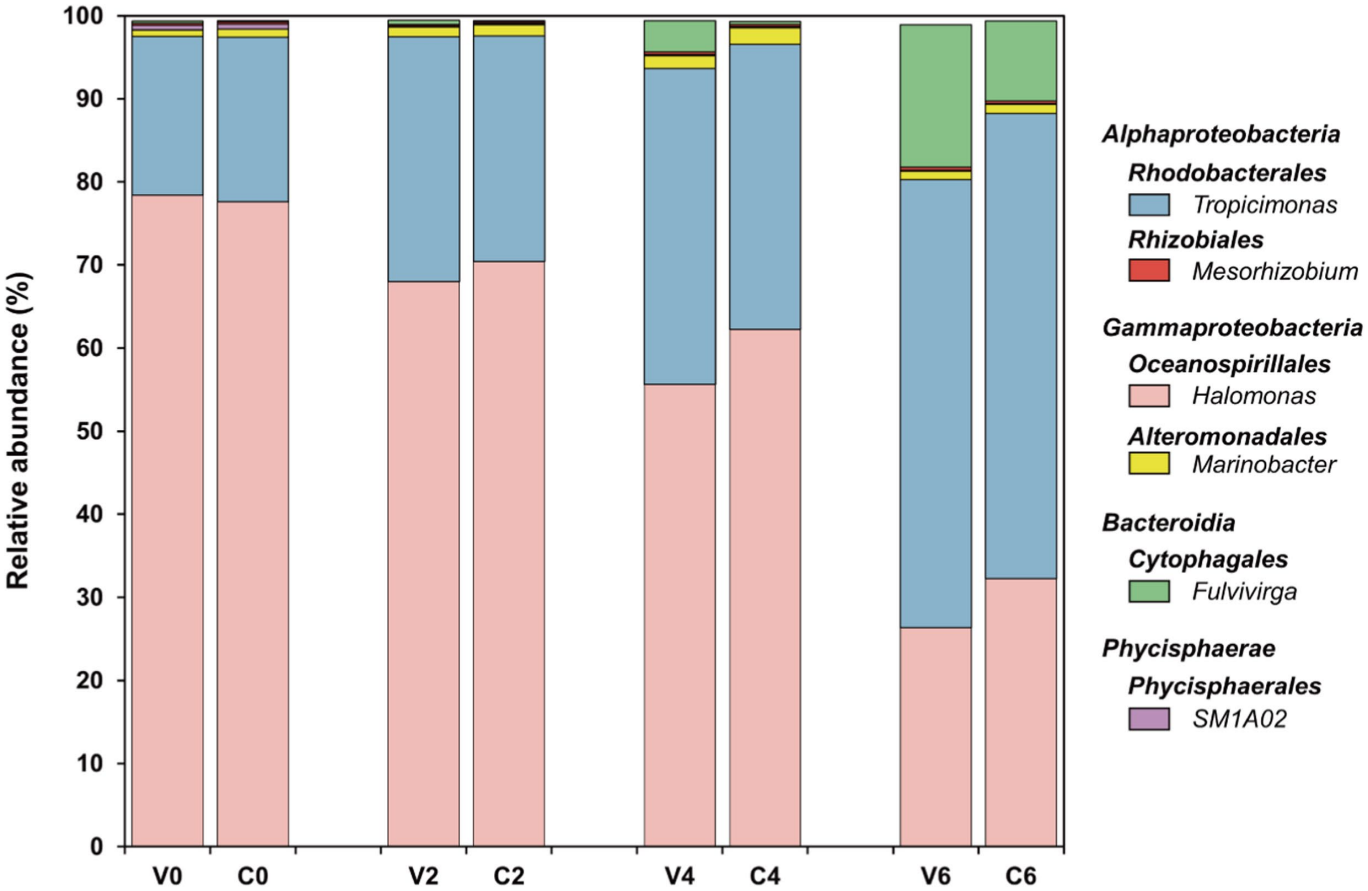
Bacterial community at the genus level in both groups over the incubations. The top five abundant genera in at least one sample were selected.

In both cyanophage-added and control cultures, the dominant taxa were *Alphaproteobacteria*, *Gammaproteobacteria*, and *Bacteroidia* (Figure 5, Supplementary Figure S4), which is congruent with those in natural seawaters (Abell and Bowman, 2005). In comparison to the control, the cyanophage-added group exhibited a lower relative abundance of *Gammaproteobacteria* throughout the incubation (Figure 5, Supplementary Figure S4). This decrease was particularly evident on day 4, when the relative abundance of *Gammaproteobacteria* in the cyanophage-added group was 7.03% less than that in the control, suggesting that substances generated in the cyanophage-added group were less favorable to the growth of *Gammaproteobacteria*. In contrast, the cyanophage-added group had a higher relative abundance of *Bacteroidia* compared to the control during the incubation (Figure 5, Supplementary Figure S4). In addition, the difference in *Bacteroidia* relative abundance between the cyanophage-added and the control cultures gradually increased as the virus-induced cell lysis augmented. On day 6, the relative abundance of *Bacteroidia*, a member of the *Cytophaga*-*Flavobacteria*-*Bacteroides* (CFB) group, exhibited a 7.91% increase in the cyanophage-added group compared to the control group, indicating that viral lysis positively influenced the growth of *Bacteroidia*. This observation aligns with previous studies that have demonstrated a similar increase in the occurrence of another CFB member, *Flavobacteria*, following the introduction of *Synechococcus*-viral lysates to a coastal bacterial community (Zhao et al., 2019) and the viral lysis of *Synechococcus* in a *Synechococcus*–heterotroph coculture (Zheng et al., 2021b). This phenomenon may be explained by the preference of the CFB group for large and complex organic matters that are rich in viral lysates (Elifantz et al., 2005). The difference in *Alphaproteobacteria* between the two groups was not as significant as those observed in the *Gammaproteobacteria* and *Bacteroidia* (Figure 5, Supplementary Figure S4). The relative abundance of *Alphaproteobacteria* in the cyanophage-added culture was higher than that in the control on day 2 and 4. The increased prevalence of *Alphaproteobacteria* in the context of cyanophage infection can be attributed to their diverse metabolic capacities. *Alphaproteobacteria* is able to assimilate and exploit biopolymers via hydrolases and can also directly utilize organic matter of low molecular weight (Zheng et al., 2018; Xie et al., 2020). The diverse origins of DOM in the coculture during cyanophage infection encompassed exudates released by both uninfected and infected *Synechococcus* cells, as well as viral lysates. Upon entry into the host cells, viruses redirect the host metabolism through the expression AMGs, resulting in alterations of the intracellular substances and the released DOM (Thompson et al., 2011; Rosenwasser et al., 2016; Jacobson et al., 2021; Kieft et al., 2021). Notably, a larger number of *Synechococcus* cells were infected and lysed on day 4 in comparison to day 2. More diverse DOM on day 4 enhanced the competitive advantage of *Alphaproteobacteria* due to their versatile metabolic capacities.

Six genera constituted the top five abundant genera in all samples, including *Tropicimonas*, *Mesorhizobium*, *Fulvivirga*, *Halomonas*, *Marinobacter*, and *SM1A02*, which belong to *Rhodobacterales* and *Rhizobiales* of *Alphaproteobacteria*, *Cytophagales* of *Bacteroidia*, *Oceanospirillales* and *Alteromonadales* of *Gammaproteobacteria*, and *Phycisphaerales* of *Phycisphaerae*, respectively (Figure 5). These six genera made up approximately 99% of the entire bacterial community in this study (Supplementary Table S1). In both the cyanophage-added and the control groups, *Tropicimonas* and *Halomonas* comprised over 80% of the overall relative abundance, with *Fulvivirga* gradually increasing to about 10–20% and eventually emerged as the dominant genus toward the later phases of the experiment. Specifically, the relative abundance of *Tropicimonas* in the cyanophage-added group was higher than the control on day 2 and 4, particularly on day 4 when the difference was 3.69%. Given the higher proportion of infected *Synechococcus* cells on day 2 and 4, the rises of *Tropicimonas* in phase I and II may benefit from the exudates of the increasingly infected *Synechococcus* cells. The relative abundance of *Mesorhizobium* in the cyanophage-added group was lower than the control in phase I, but exceeded the control on days 4 and 6 with an increasing difference, indicating that increasing viral lysates in phase II and III facilitated the growth of *Mesorhizobium*. The relative abundance of *Fulvivirga* in the cyanophage-added group was higher than that in the control, particularly on day 6 when the difference reached 7.50%, aligning with the preference of *Bacteroides* for large and complex organic matters (Elifantz et al., 2005) which may be rich in viral lysates of phase III. In contrast to *Fulvivirga*, the relative abundances of *Halomonas* and *Marinobacter* in the cyanophage-added group were lower than those in the control, with the largest difference occurring on day 4. The growth inhibition further revealed that the high amount of exudates released by infected *Synechococcus* cells in phase II were detrimental to the growth of these two *Gammaproteobacteria* genera. Moreover, the relative abundance of *SM1A02* in the cyanophage-added group consistently remained lower than that in the control, with the largest difference (0.05%) observed on day 2, indicating that DOM generated by viral infection was unfavorable for the competitiveness of *SM1A02* in the bacterial community, especially the exudates of infected *Synechococcus* cells in phase I.

Furthermore, viral infection of *Synechococcus* had a notable impact on the minor bacterial taxa at the class level as well. Specifically, the relative abundances of *Campylobacteria* and *Verrucomicrobiae* exhibited a statistically significant decrease in the cyanophage-added group compared to those in the control on day 2 and 4, respectively (Supplementary Figure S5). The prevalence of *Verrucomicrobiae* during a cyanobacterial bloom that occurred in Meiliang Bay of Lake Taihu indicates its preference for cyanobacteria-derived DOM (Shi et al., 2017). It is plausible that viral lysates or the exudates of infected *Synechococcus* cells differ significantly from exudates of uninfected *Synechococcus* cells, hindering the growth of *Campylobacteria* and *Verrucomicrobiae* and leading to a decline in their relative abundances following cyanophage introduction.

### Effects of cyanophage infection on the interaction among bacterial taxa

**Figure 6:**
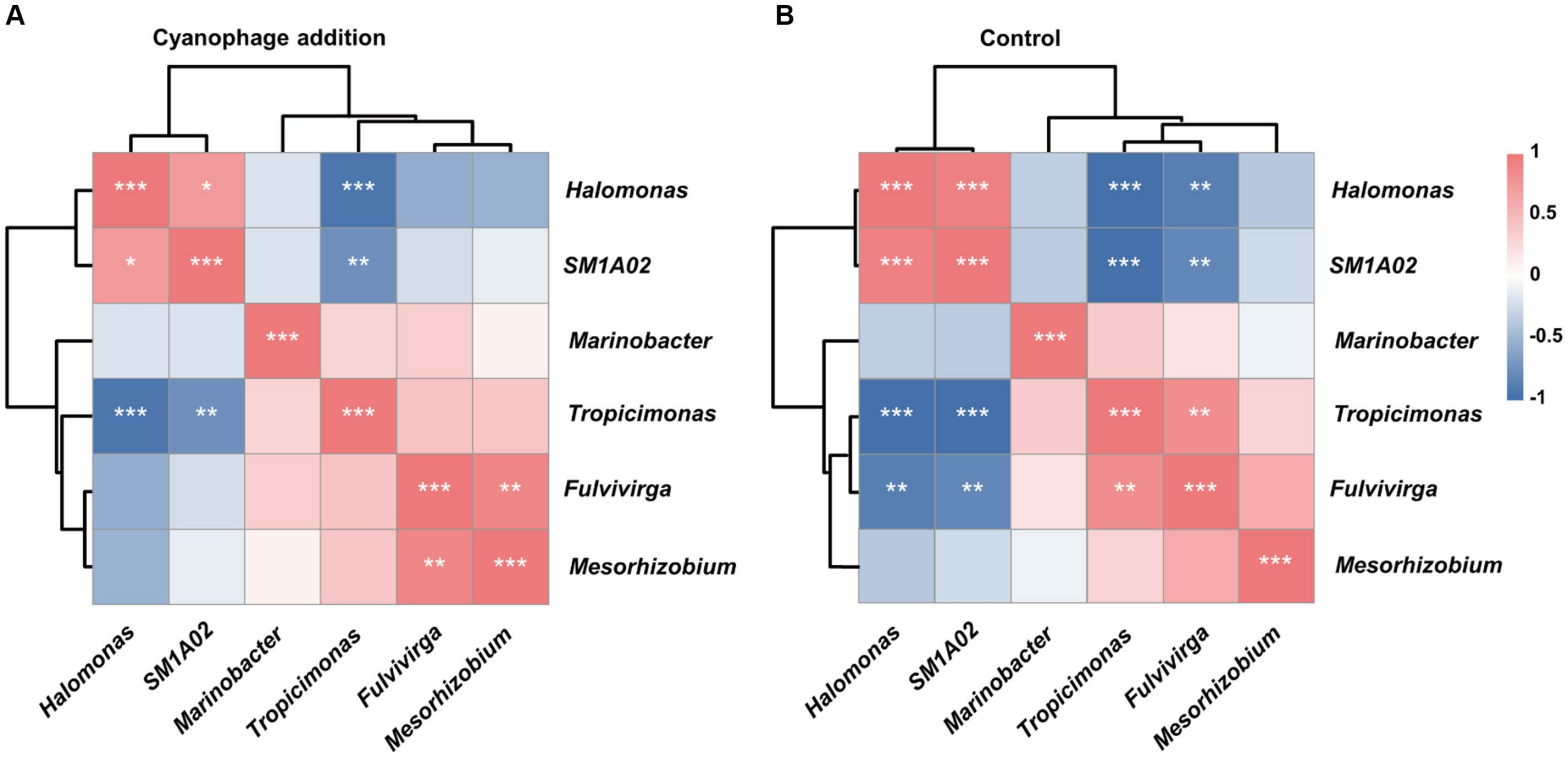
Clustering Spearman correlation heatmaps for the top six genera in the cyanophage-added **(A)** and the control **(B)** groups. Level of significance: *p < 0.05; **p < 0.01; ***p < 0.001.

The correlation analysis between the relative abundances of the top six genera in the cyanophage-added and the control groups revealed a positive correlation between *Halomonas* and *SM1A02*, but both of these two genera exhibited a negative correlation with the remaining genera (Figure 6). A majority of the other genera displayed a positive correlation with one another. Viral infection of *Synechococcus* did not change the correlations among the top six genera, but affected the significance of correlations between certain genera (Figure 6). Specifically, the cyanophage infection weakened the positive correlations between *Halomonas* and *SM1A02*, *Tropicimonas* and *Fulvivirga*, and the negative correlations among *Halomonas*, *SM1A02*, and *Fulvivirga*, but enhanced the positive correlation between *Mesorhizobium* and *Fulvivirga*. The alterations in the relationship between dominant genera can potentially be ascribed to the variation of DOM caused by viral infection, which can be further illustrated by examining the dynamics and variations in DOM molecular composition during viral infection as well as the correlations between the DOM components and each genus.

**Figure 7:**
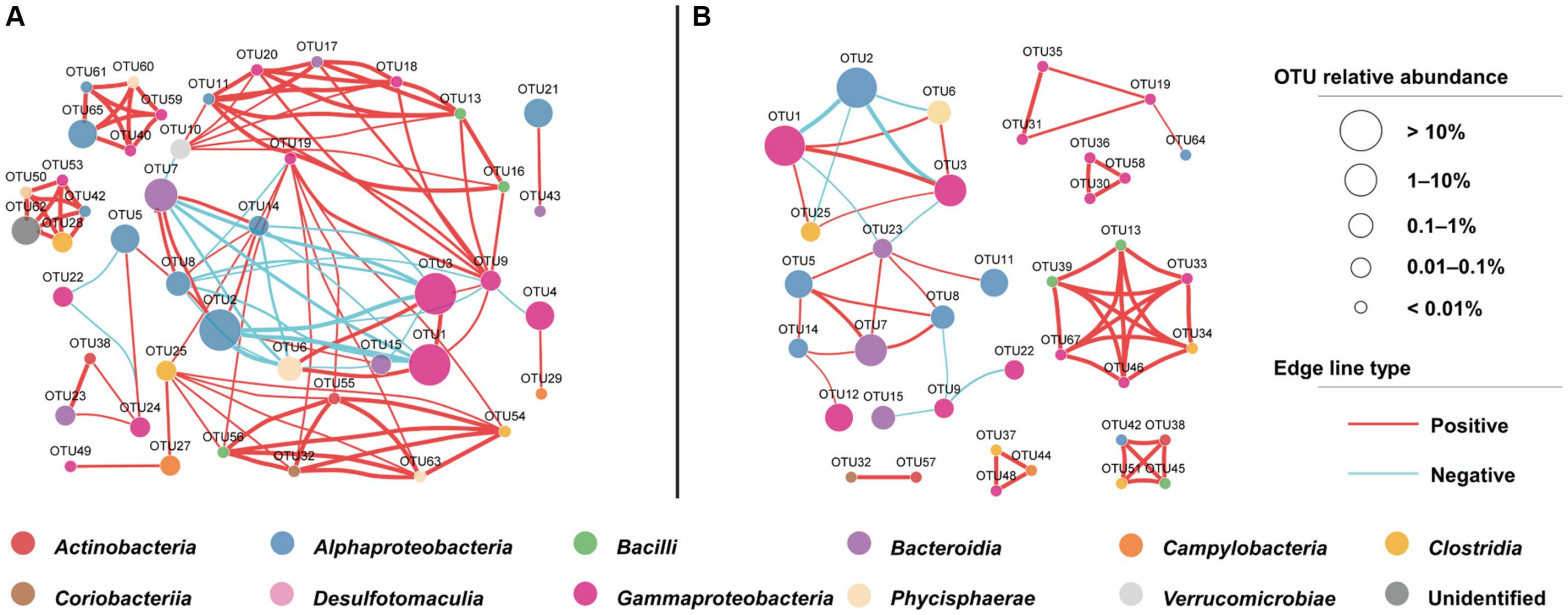
OTU-based network uncovering different interactions among bacterial taxa under the cyanophage infection. The control network **(A)**, the cyanophage-added network **(B)**.

The microbial cooccurrence network analysis based on OTUs revealed that the network characteristics of the cyanophage-added and the control groups were significantly different. The cyanophage-added network exhibited a smaller number of nodes and edges, comprising 37 nodes and 56 edges, in comparison to the control network which comprised 44 nodes and 109 edges (Supplementary Table S2). The clustering coefficient and the average number of neighbors of the cyanophage-added network were also considerably lower than those in the control network (Figure 7). The clustering coefficient acts for the network complexity and robust interactions between microorganisms (Guo et al., 2022), and the higher the clustering coefficient, the higher the dynamics and activity of the community (de Vries et al., 2018). The clustering coefficient in the cyanophage-added group was significantly lower than that in the control, suggesting that viral infection of *Synechococcus* reduced the overall microbial interactions.

In both cyanophage-added and control groups, a majority of highly connected OTUs were classified into classes *Alphaproteobacteria* and *Gammaproteobacteria* (Figure 7). This result was consistent with the impact of the *Prochlorococcus* viral lysates on the bacterial community in the oligotrophic South China Sea (Xiao et al., 2021), emphasizing that connections between microbial groups primarily occur among dominant taxa. However, there were notable differences in the highly connected OTUs between the cyanophage-added and the control groups. In the control, the top five highly connected OTUs belonged to classes *Gammaproteobacteria* and *Alphaproteobacteria*. In the cyanophage-added group, the top five highly connected OTUs belonged to *Bacilli*, *Gammaproteobacteria*, and *Clostridia*. The relative abundance of OTUs belonging to *Bacilli* and *Clostridia* in the cyanophage-added group was lower than that of *Gammaproteobacteria* and *Alphaproteobacteria*, suggesting that cyanophage infection may decrease interactions among highly abundant bacterial taxa and promote interactions among less abundant taxa (Supplementary Table S3). Additionally, the cyanophage-added group exhibited a greater proportion of positive to negative microbial interactions than the control (Supplementary Table S2). The decline in the ratio of negative interactions in the cyanophage-added group was mainly due to specific OTUs belonging to *Alphaproteobacteria* (OTU5, OTU14), *Bacteroidia* (OTU7), and *Gammaproteobacteria* (OTU19). These OTUs demonstrated negative interactions with other OTUs in the control, but not in the cyanophage-added group, indicating that viral infection diminished the competitive pressure among specific heterotrophic bacterial taxa.

### Coupling between FDOM components, nutrients and bacterial community under viral infection of *Synechococcus*

**Figure 8:**
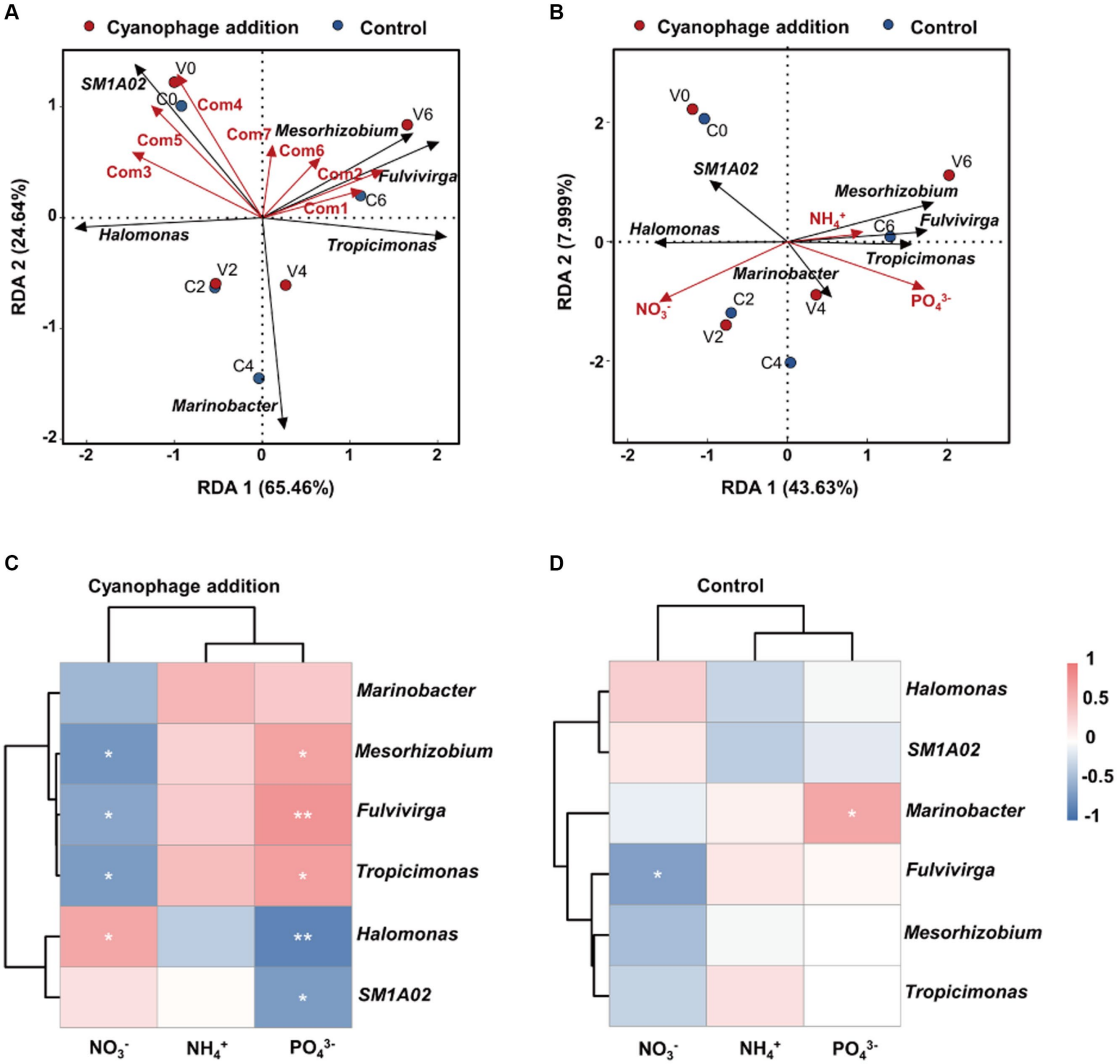
Redundancy analysis of dominant genera with the FDOM components **(A)** and nutrient factors **(B)**. Clustering Spearman correlation heatmaps for the top six genera and nutrients in the cyanophage-added **(C)** and the control **(D)** groups. Level of significance: *p < 0.05; **p < 0.01; ***p < 0.001.

The relative abundances of the top six genera showed varying correlations with FDOM components and nutrients according to the RDA analysis (Figures 8A,B). *Fulvivirga* and *Mesorhizobium* demonstrated positive correlations with Com1, 2, 6 and 7, while displaying negative correlations with Com3 and 5. *Tropicimonas* exhibited positive correlations with Com1, 2, and 6, but negative correlations with Com3–5, which contrasted with the manner of *Halomonas*. *SM1A02* displayed positive correlations with Com3–5 and 7, but negative correlations with Com1 and 2. *Marinobacter* exhibited negative correlations with all FDOM components. In the cyanophage-added culture, higher relative abundances of *Fulvivirga* and *Mesorhizobium*, and lower relative abundances of *Halomonas* compared to the control in phases II and III were coupling with the gradually increased fluorescence intensity of Com 6 and 7, indicating that DOM produced from viral infection of *Synechococcus* were potentially important factors in influencing the growth of specific surrounding heterotrophic bacteria. Moreover, *Tropicimonas*, *Mesorhizobium*, *Fulvivirga*, and *Marinobacter* exhibited a positive correlation with ammonium and phosphate, and a negative correlation with nitrate, while *Halomonas* and *SM1A02* displayed the opposite trend (Figure 8B). Viral infection of *Synechococcus* generally enhanced the correlation between the top six genera and nutrients, regardless of whether it was positive or negative (Figures 8C,D). However, the significant positive correlation between *Marinobacter* and phosphate was observed in the control, but not in the cyanophage-added group.

The enhanced or weakened correlations between the top genera and nutrients can be attributed to the alterations of the inorganic and organic matter by cyanophage infection within the culture system and different metabolic potentials of specific bacterial taxa. Decreased nitrate in phase III of the cyanophage-added cultures would be unfavorable for the growth of *Halomonas* which would benefit from denitrification, an important trait of this genus (González-Domenech et al., 2010; Wang and Shao, 2021). In addition, cyanophage infection could change the DOM composition in the culture. After entry into the host cells, cyanophages would change the host metabolism by expressing AMGs related to carbon, nitrogen, and phosphorus metabolisms (Thompson et al., 2011; Roux et al., 2016; Jacobson et al., 2021), which leads to element changes of both form and composition within host cells and subsequently makes the released DOM different from the uninfected cell exudates. Viral lysates comprise a wide range of complex and diverse DOM and possess elemental ratios that differ from those in the surrounding environment (Jover et al., 2014). Studies have shown that cyanophage lysates are rich in CHON compounds and are an important source of dissolved organic nitrogen in the marine environment (Ma et al., 2018; Zhao et al., 2019; Zheng et al., 2021a). Furthermore, virus particles, consisting of protein capsid and nucleic acid, are rich in nitrogen and phosphorus and also serve as DOM constituents that could be utilized by the surrounding bacterial community (Fuhrman, 1999). DOM alterations caused by cyanophage infection may stimulate different metabolisms of specific bacterial taxa from those in the control, resulting in changes in correlations between the bacterial taxa and nutrients in the cyanophage-added system. Furthermore, diverse metabolic activities of the bacterial taxa belonging to *Alphaproteobacteria* (such as *Tropicimonas*, *Mesorhizobium*) and *Bacteroidia* (especially *Fulvivirga*) may enable the mineralization of cyanophage-amended DOM and the subsequent accumulation of ammonium and phosphate within the system.

### Viral infection of *Synechococcus* may change the metabolism of the microbial community

**Figure 9:**
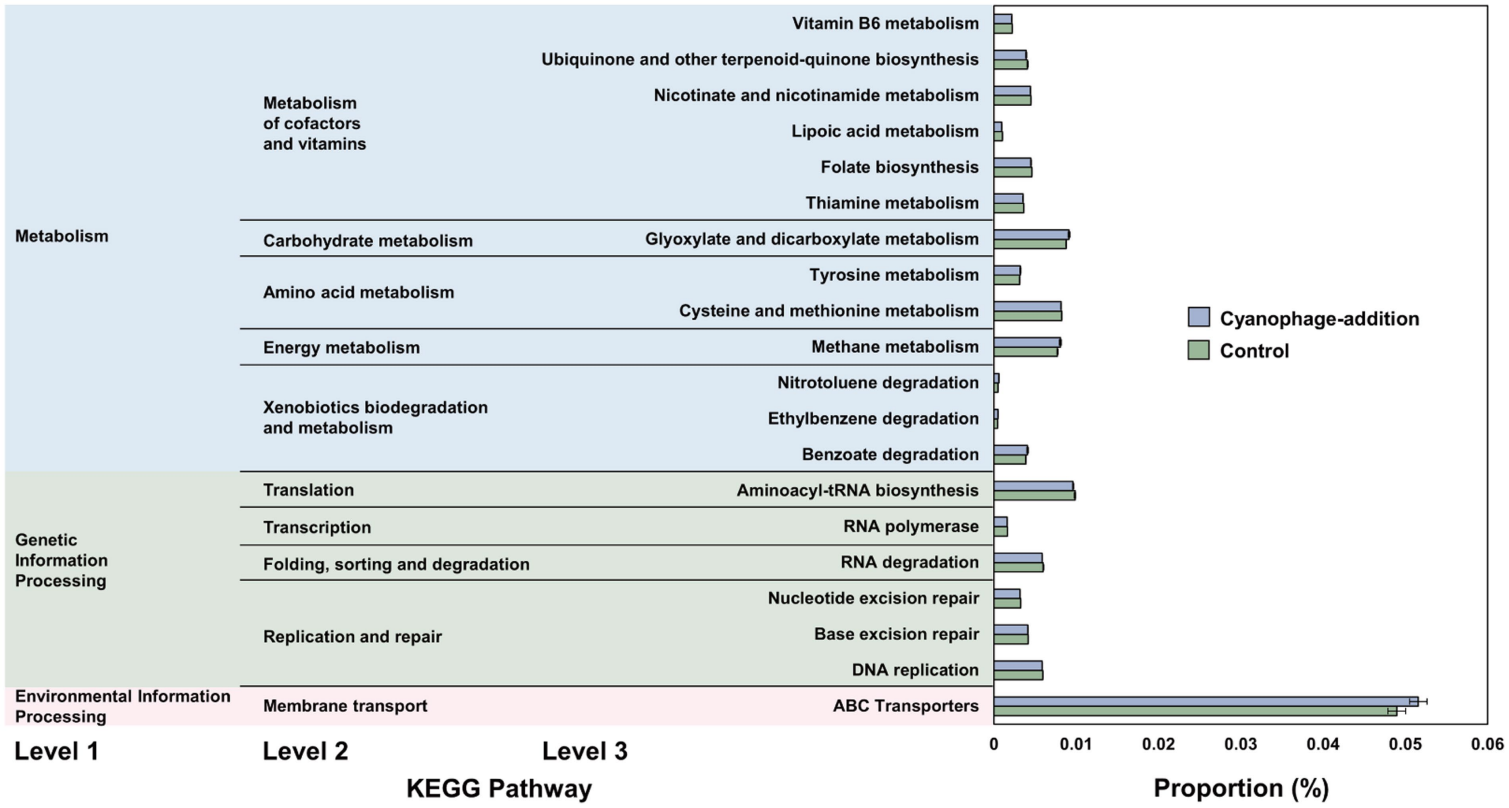
Comparison of the proportions of KEGG functional profiles between the cyanophage-added and the control cultures on day 4. Only differences with a *p*-value less than 0.05 are shown.

The transition of the bacterial community may cause the variation of the whole metabolism of the bacterial community. PICRUSt analysis was carried out to investigate the potential influence of cyanophage infection on the function of the microbial community. Statistically significant differences in potential metabolic functions were only observed between the cyanophage-added and the control groups on day 4 when a high proportion of cyanophage-infected *Synechococcus* cells were present in the cyanophage-added culture. In the second-tier KEGG pathway level, the cyanophage-added group exhibited a higher proportion than the control within KEGG categories of “carbohydrate metabolism,” “energy metabolism,” “xenobiotics biodegradation and metabolism,” and “membrane transport” (Figure 9), but displayed less proportion of functional traits crucial for typical cellular growth and metabolism than the control, including pathways classified as “metabolism of cofactors and vitamins,” “translation,” “folding, sorting and degradation,” and “replication and repair.” In the third-tier level, the cyanophage-added group displayed a higher proportion in various KEGG pathways than the control, such as tyrosine metabolism, glyoxylate and dicarboxylate metabolism, methane metabolism, ABC transporters, transcription factors, and xenobiotics (nitrotoluene, ethylbenzene, and benzoate) biodegradation. Conversely, proportions of some functions were lower in the cyanophage-added group than in the control, such as aminoacyl-tRNA biosynthesis, RNA degradation, DNA replication, nucleotide excision repair, cysteine and methionine metabolism, vitamin B6 metabolism, and lipoic acid metabolism, etc. (Figure 9).

Among all KEGG pathways, the most affected by cyanophage infection was “ABC transporters.” Transporters are extremely important for the survival of bacteria by helping bacterial cells absorb nutrients and resist various internal and external pressures (Piepenbreier et al., 2017). Diverse DOMs consisting of viral lysates, exudates from infected and uninfected *Synechococcus* cells in the cyanophage-added cultures on day 4 may be responsible for the significant differences of transporters from those in the control. Notably, the proportion of the “methane metabolism” pathway on day 4 was significantly higher than those on day 0 and day 2 in the cyanophage-added group (Supplementary Figure S6), indicating that exudates released by the high proportion of viral infected *Synechococcus* cells on day 4 may contribute to stimulate growth of bacterial populations related to methane metabolism. It has been reported that *Synechococcus* is capable of producing methane (Gomez-Garcia et al., 2011; Kutovaya et al., 2013; Bižić et al., 2020), a strong greenhouse gas with a global warming potential 25 times greater than that of CO_2_ in 100 years (Hao et al., 2021). The significant difference in the methane-metabolism potentials observed among bacterial communities in the cyanophage-added and the control groups suggests that cyanophage infection has the potential to impact the production of methane-related compounds. This finding holds significant implications for global climate change and biogeochemical cycles, considering the numerous cyanophage infections occurring in marine ecosystems. Furthermore, the significantly higher relative abundance of “xenobiotics degradation and metabolism” pathway in the cyanophage-added group indicates that viral infection of *Synechococcus* may result in increased levels of aromatic substances (here benzoate, ethylbenzene, and nitrotoluene) in the cultures. In addition, the relative abundance of tyrosine (an aromatic amino acid) metabolism in the cyanophage-added group was also higher than that in the control. Given the lower bioavailability of aromatic compounds (Xie et al., 2020), increased metabolic potential on aromatic compounds of the surrounding bacterial community indicates that infected *Synechococcus* cells may contribute to the production of refractory DOMs in the ocean.

## Conclusion

This study demonstrated that viral infection of *Synechococcus* has a significant impact on the co-occurring heterotrophic bacterial community, influencing its dynamics, diversity, composition, interactions and predicted metabolic functions. It is intriguing to find that cyanophage infection reduced the bacterial interactions, but enhanced the correlation between bacterial taxa and nutrients. Moreover, cyanophage infection altered DOM compositions and nutrient dynamics and may contribute to the production of methane-related compounds and refractory organic matters in the coculture system, which have great implications for global climate change and biogeochemical cycles in the ocean. Notably, the exudates of viral-infected *Synechococcus* cells exhibited distinct effects on the co-occurring heterotrophic bacterial community from the viral lysates and facilitated to increase the diversity and change metabolic potentials of the bacterial community. This observation further illustrated the hypothesis that viral infection in the ocean is a continuous process that has implications for the surrounding environment and biogeochemistry beyond the simple release of viral lysates. Further efforts are needed to illustrate the DOM composition of the *Synechococcus* virocell exudates and its transformation by the co-occurring heterotrophic bacterial community. Viral interference on the methane or its derivatives production of *Synechococcus* cells is also worthy of further study.

## Data availability statement

The datasets presented in this study can be found in online repositories. The names of the repository/repositories and accession number(s) can be found in the article/Supplementary material.

## Author contributions

HM: Data curation, Writing – original draft, Investigation, Methodology. BL: Investigation, Methodology, Writing – original draft. HZ: Data curation, Writing – original draft. JL: Writing – review & editing, Funding acquisition, Supervision. YX: Writing – review & editing, Conceptualization, Supervision. FC: Writing – review & editing, Conceptualization, Funding acquisition, Supervision.

## Footnotes

^1^ https://cshprotocols.cshlp.org/content/2022/10/pdb.rec108001.short

^2^ http://www.cloudtutu.com

^3^ https://www.omicstudio.cn/tool
