# Supplementary material for: Viral infection of an estuarine Synechococcus influences its co-occurring heterotrophic bacterial community in the culture
Source: Front Microbiol. 2024 Jan 25;15:1345952. doi: 10.3389/fmicb.2024.1345952 (PMC10854222; doi:10.3389/fmicb.2024.1345952)
Supplement: Supplementary file 1 [file Data_Sheet_1.docx]

Supplementary Material

Viral infection of an estuarine *Synechococcus* influences its co-occurring heterotrophic bacterial community in the culture

**The Supporting Information contains:**

**Table S1.** The relative abundances of the top six OTUs.

**Table S2.** Summary of the OTU-based network.

**Table S3.** The top five highly connected OTUs in the network.

**Figure S1.** Rarefaction curves based on the OTU richness.

**Figure S2.** Three distinct phases of cyanophage infection in the *Synechococcus*–heterotrophic bacteria coculture system.

**Figure S3.** EEM fluorescence spectra of the DOM in the cyanophage-added (top) and the control (bottom) cultures during the incubation.

**Figure S4.** Bacterial community at the class level in both groups over the incubations.

**Figure S5.** Significant different relative abundances of *Campylobacteria* on day 2 (*P* = 0.04) and *Verrucomicrobiae* on day 4 (*P* = 0.04) between the cyanophage-added and control groups.

**Figure S6.** Comparison of the predicted methane metabolism pathway proportions of the heterotrophic bacterial community in the cyanophage-added group. ***P* < 0.01.

Table S1. The relative abundances of the top six OTUs.

| Genus | Relative abundance (%) | | | | | | | |
| --- | --- | --- | --- | --- | --- | --- | --- | --- |
|  | V0 | C0 | V2 | C2 | V4 | C4 | V6 | C6 |
| *Tropicimonas* | 19.08 | 19.84 | 29.46 | 27.15 | 38.02 | 34.33 | 53.92 | 55.95 |
| *Mesorhizobium* | 0.26 | 0.28 | 0.19 | 0.22 | 0.30 | 0.29 | 0.39 | 0.32 |
| *Halomonas* | 78.43 | 77.61 | 68.03 | 70.43 | 55.62 | 62.24 | 26.35 | 32.26 |
| *Marinobacter* | 0.78 | 0.98 | 1.17 | 1.33 | 1.55 | 1.98 | 1.05 | 1.12 |
| *Fulvivirga* | 0.27 | 0.15 | 0.52 | 0.14 | 3.82 | 0.36 | 17.13 | 9.63 |
| *SM1A02* | 0.57 | 0.59 | 0.10 | 0.15 | 0.13 | 0.13 | 0.08 | 0.08 |

Table S2. Summary of the OTU-based network.

|  | Control | Cyanophage-added |
| --- | --- | --- |
| Edge | 109 | 56 |
| Node | 44 | 37 |
| Average number of neighbors | 5.50 | 3.20 |
| Positive/Negative | 87/22 | 47/9 |
| Negative ratio (%) | 20.18 | 16.07 |
| Clustering coefficient | 0.70 | 0.43 |
| Characteristic path length | 2.89 | 2.69 |
| Network density | 0.18 | 0.23 |
| Network centralization | 0.22 | 0.23 |

Table S3. The top five highly connected OTUs in the network.

| Control | | | Cyanophage-added | | |
| --- | --- | --- | --- | --- | --- |
| OTU | Class | Degree | OTU | Class | Degree |
| OTU9 | *Gammaproteobacteria* | 12 | OTU23 | *Bacteroidia* | 6 |
| OTU8 | *Alphaproteobacteria* | 9 | OTU39 | *Bacilli* | 5 |
| OTU19 | *Gammaproteobacteria* | 8 | OTU13 | *Bacilli* | 5 |
| OTU3 | *Gammaproteobacteria* | 8 | OTU34 | *Clostridia* | 5 |
| OTU1 | *Gammaproteobacteria* | 8 | OTU1 | *Gammaproteobacteria* | 5 |


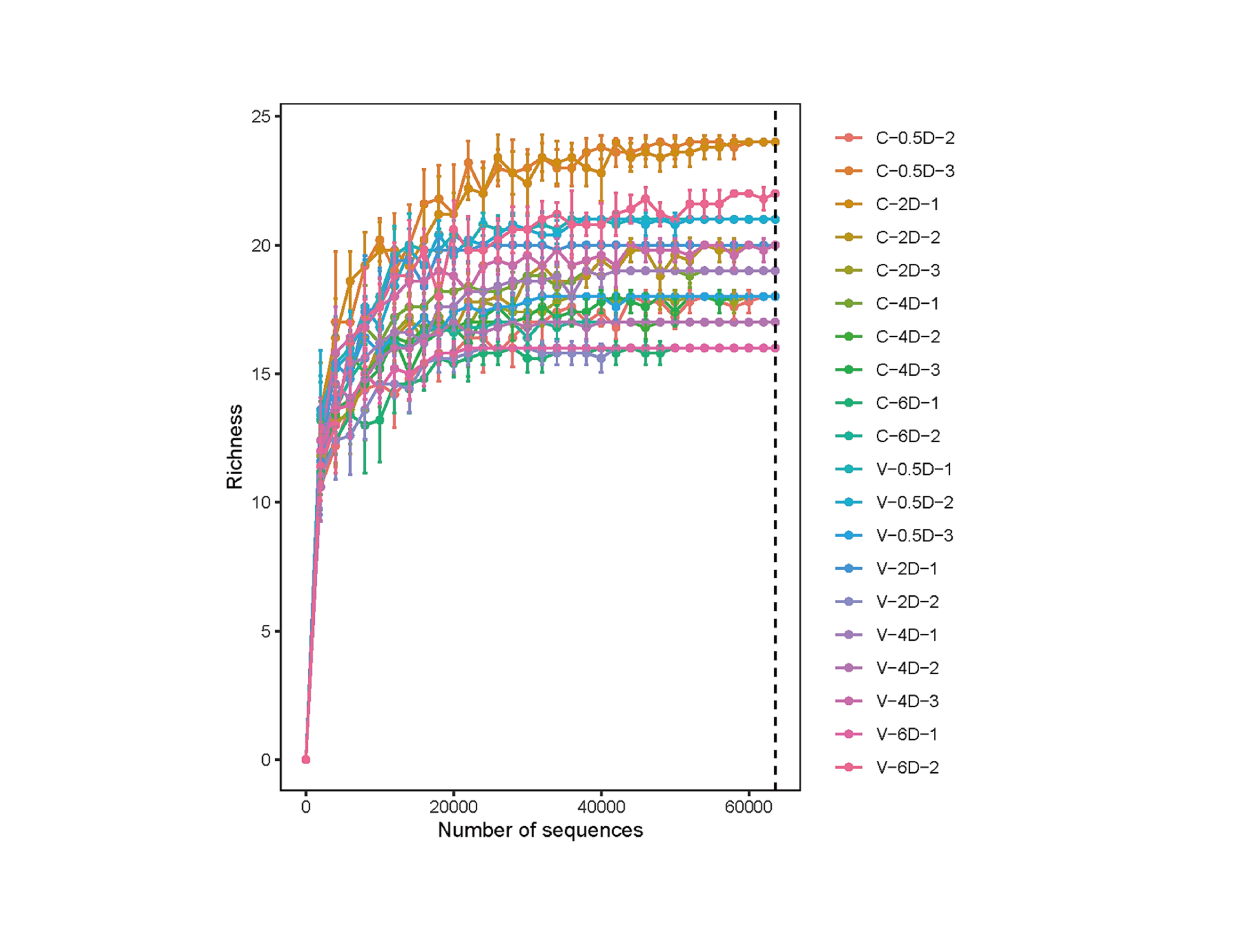


Figure S1. Rarefaction curves based on the OTU richness.


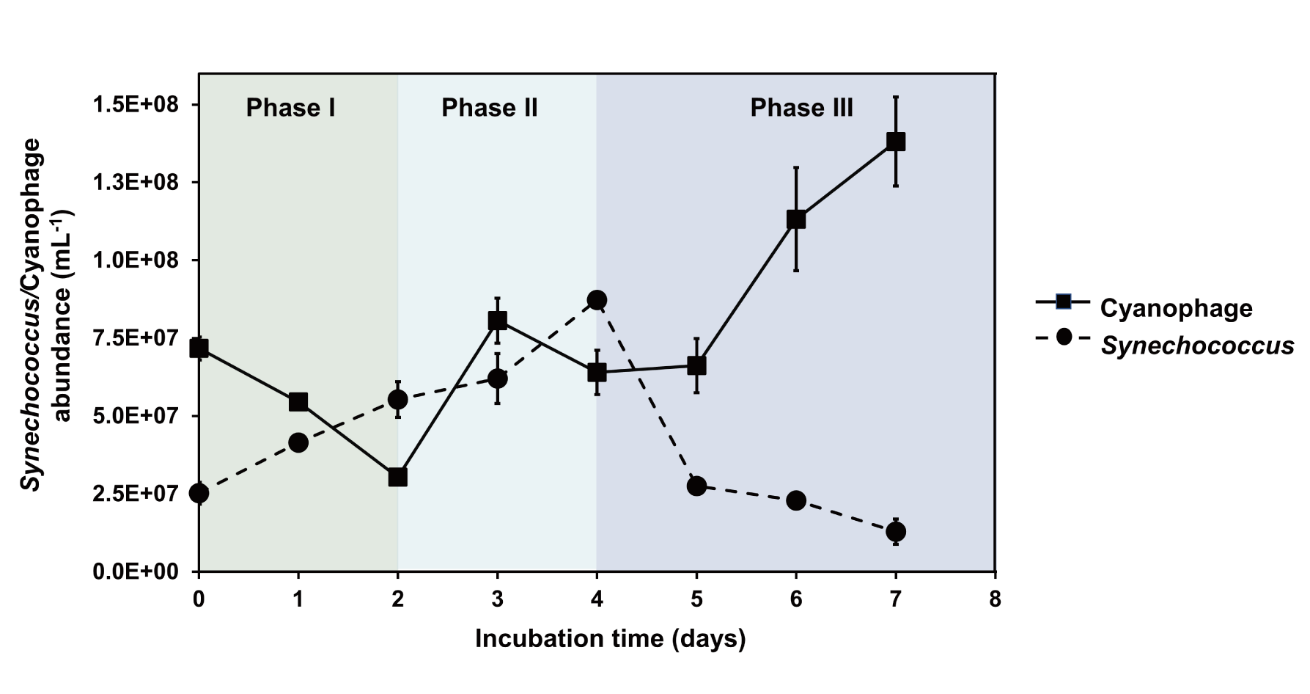


Figure S2. Three distinct phases of cyanophage infection in the *Synechococcus*–heterotrophic bacteria coculture system.


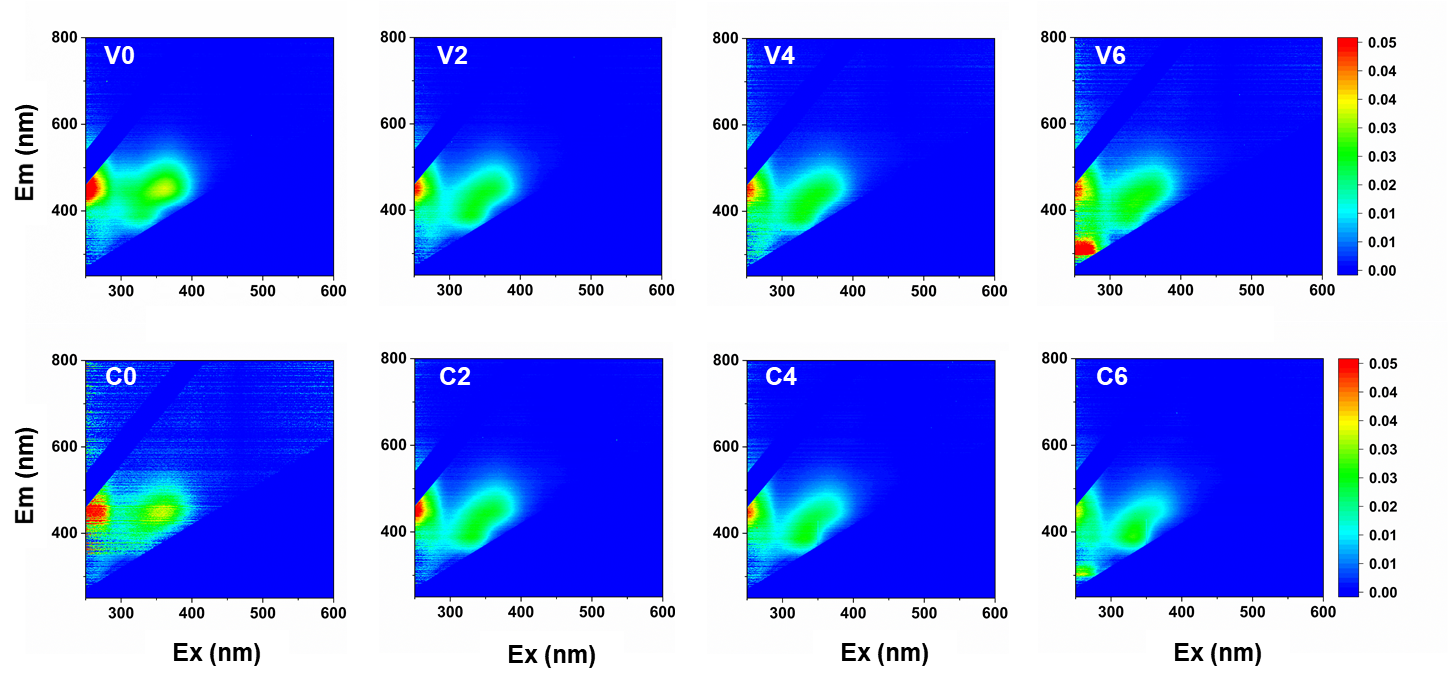


Figure S3. EEM fluorescence spectra of the DOM in the cyanophage-added (top) and the control (bottom) cultures during the incubation.


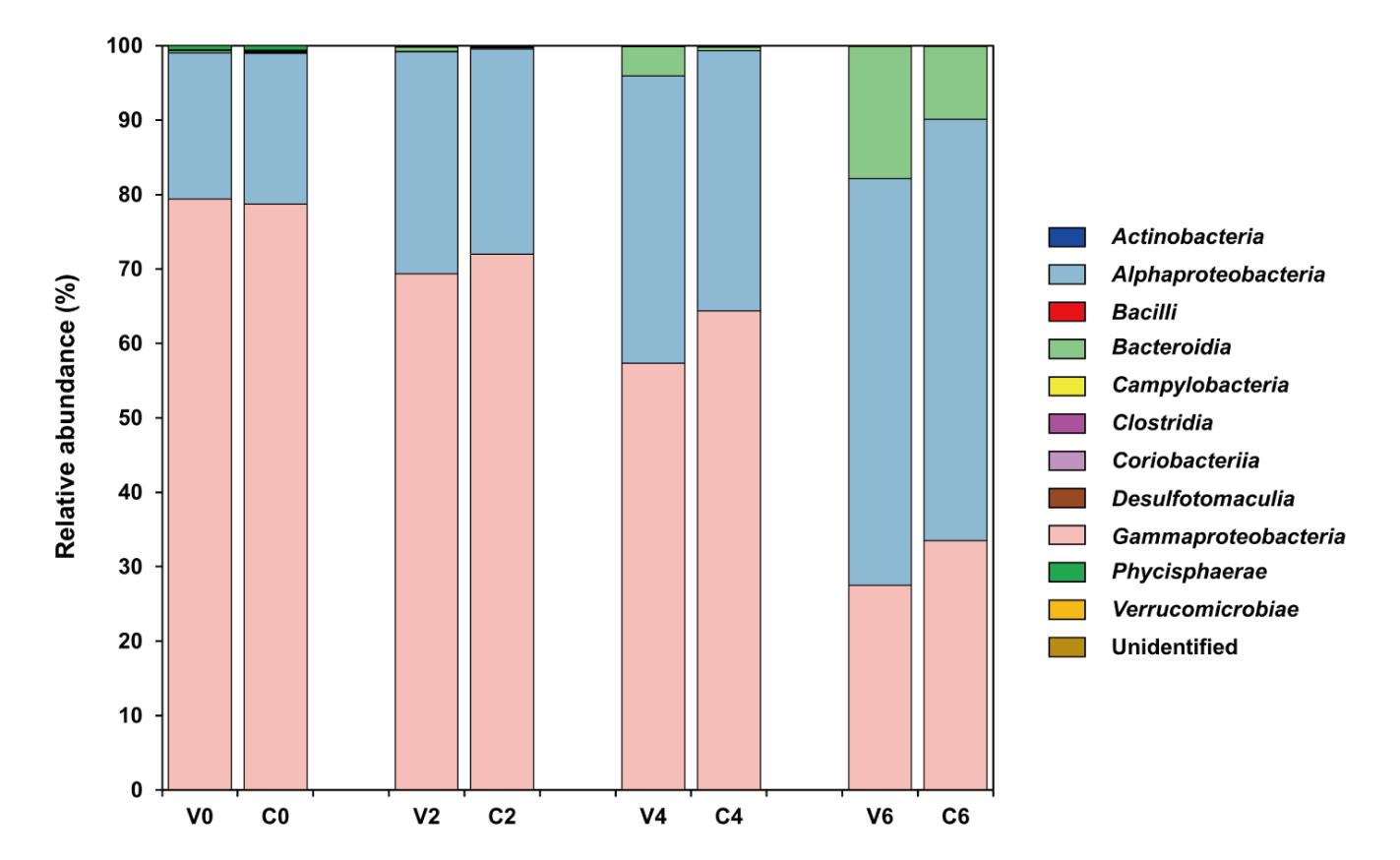


Figure S4. Bacterial community at the class level in both groups over the incubations.


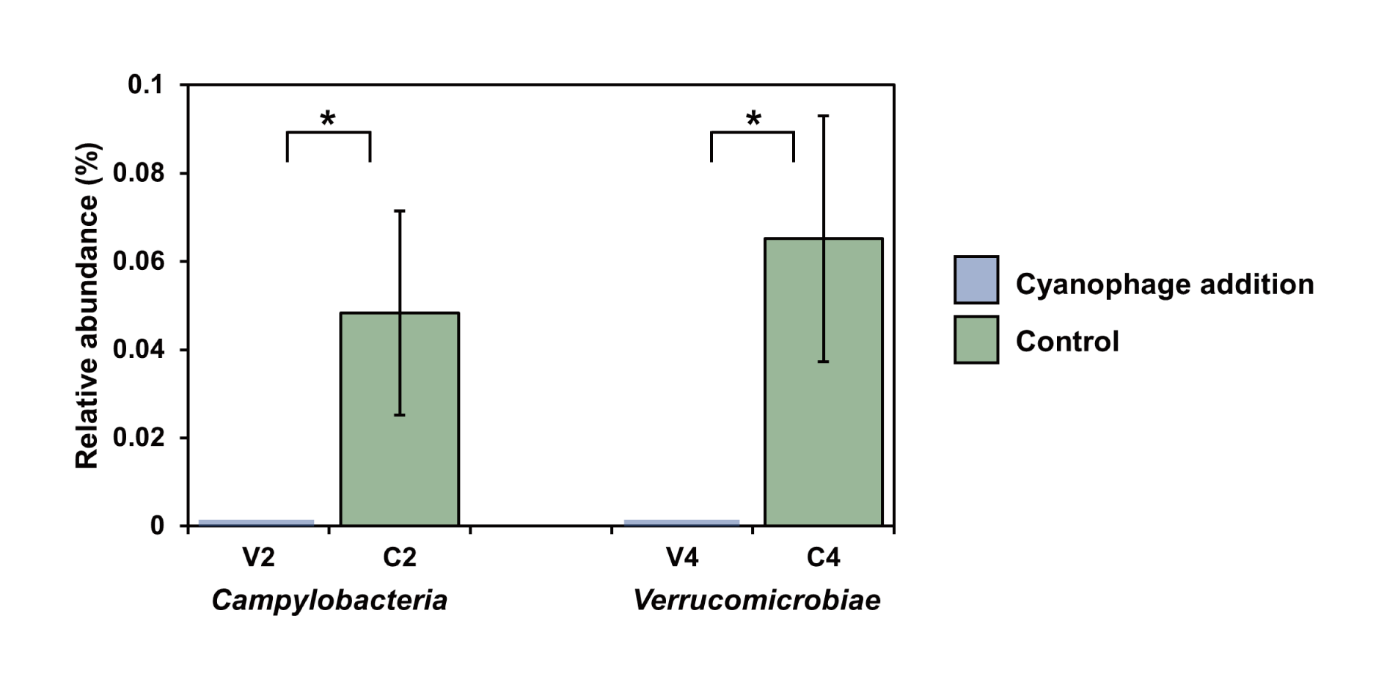


Figure S5. Significant different relative abundances of *Campylobacteria* on day 2 (*P* = 0.04) and *Verrucomicrobiae* on day 4 (*P* = 0.04) between the cyanophage-added and control groups.


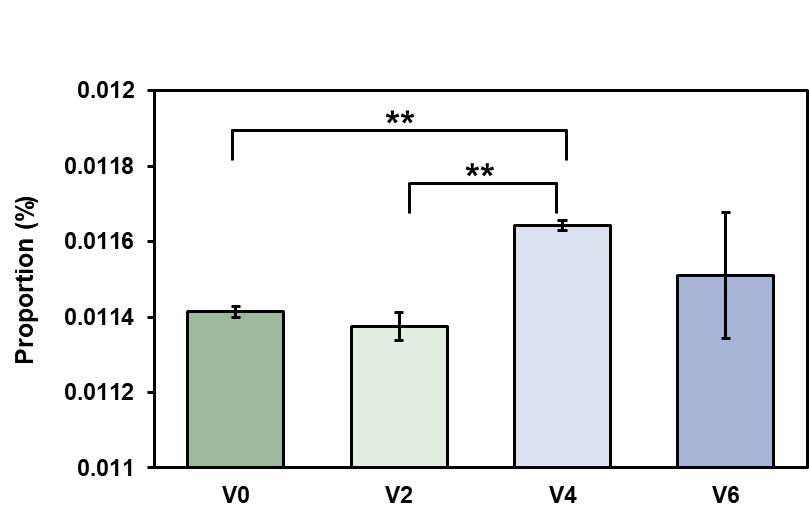


Figure S6. Comparison of the predicted methane metabolism pathway proportions of the heterotrophic bacterial community in the cyanophage-added group. ***P* < 0.01.
